# Comparison of static and dynamic models of maternal immunization to prevent infant pertussis in Brazil

**DOI:** 10.1016/j.vaccine.2020.09.006

**Published:** 2021-01-03

**Authors:** Louise B. Russell, Sun-Young Kim, Cristiana Toscano, Ben Cosgriff, Ruth Minamisava, Ana Lucia Andrade, Colin Sanderson, Anushua Sinha

**Affiliations:** a University of Pennsylvania, Department of Medical Ethics and Health Policy, c/o Lauren Counterman, 423 Guardian Drive, Philadelphia, PA 19104, USA; b Seoul National University, Department of Public Health Sciences, Graduate School of Public Health, 1 Gwanak-ro, Gwanak-gu, Seoul 08826, South Korea; c Institute of Tropical Pathology and Public Health, Federal University of Goiás, Goiânia, Goiás, Brazil; d Consultant, Westfield, NJ 07090, USA; e School of Nursing, Federal University of Goiás, Goiânia, Goiás, Brazil; f London School of Hygiene and Tropical Medicine, Department of Health Services Research and Policy, 15-17 Tavistock Place, London WC1H 9SH, United Kingdom; g Department of Health Systems and Policy, School of Public Health, Rutgers University, Piscataway, NJ, USA

**Keywords:** Cost-effectiveness, Dynamic transmission model, Infectious disease model, Maternal immunization, Pertussis, Static model

## Abstract

**Background:**

This paper compares cost-effectiveness results from two models of maternal immunization to prevent pertussis in infants in Brazil, one static, one dynamic, to explore when static models are adequate for public health decisions and when the extra effort required by dynamic models is worthwhile.

**Methods:**

We defined two scenarios to explore key differences between static and dynamic models, herd immunity and time horizon. Scenario 1 evaluates the incremental cost/DALY of maternal acellular pertussis (aP) immunization as routine infant vaccination coverage ranges from low/moderate up to, and above, the threshold at which herd immunity begins to eliminate pertussis. Scenario 2 compares cost-effectiveness estimates over the models’ different time horizons. Maternal vaccine prices of $9.55/dose (base case) and $1/dose were evaluated.

**Results:**

The dynamic model shows that maternal immunization could be cost-saving as well as life-saving at low levels of infant vaccination coverage. When infant coverage reaches the threshold range (90–95%), it is expensive: the dynamic model estimates that maternal immunization costs $2 million/DALY at infant coverage > 95% and maternal vaccine price of $9.55/dose; at $1/dose, cost/DALY is $200,000. By contrast, the static model estimates costs/DALY only modestly higher at high than at low infant coverage. When the models’ estimates over their different time horizons are compared at infant coverage < 90–95%, their projections fall in the same range.

**Conclusions:**

Static models may serve to explore an intervention’s cost-effectiveness against infectious disease: the direction and principal drivers of change were the same in both models. When, however, an intervention too small to have significant herd immunity effects itself, such as maternal aP immunization, takes place against a background of vaccination in the rest of the population, a dynamic model is crucial to accurate estimates of cost-effectiveness. This finding is particularly important in the context of widely varying routine infant vaccination rates globally.

**Clinical Trial registry:**

Clinical Trial registry name and registration number: Not applicable.

## Highlights

• Dynamic transmission models of infectious disease capture the herd immunity effects of vaccination.
• We compared dynamic and static models of maternal acellular pertussis (aP) immunization built with Brazilian data.
• At infant vaccine coverage < 90–95%, both models estimate that maternal immunization is cost-effective.
• Only the dynamic model shows that maternal immunization is not cost-effective at infant coverage > 90–95%.
• The background effect of routine infant vaccination is critical to the cost-effectiveness of maternal aP immunization.

## 1 Introduction

The special characteristics of many infectious diseases – transmission from one individual to others, natural immunity after recovery from the disease, and the herd immunity that protects susceptible individuals when other people in the population are immune – are best captured by dynamic transmission simulation models. Static models, which are easier, faster, and less costly to develop and implement, are acceptable in some circumstances, such as when herd immunity is not an issue [1], [2], [3], [4].

Immunization of pregnant women with a single dose of acellular pertussis (aP) vaccine to prevent pertussis in infants too young to receive their own vaccine is an intervention for which a static model might be sufficient. The target population, pregnant women, is a small part of the total population, and the period of immunity conferred on infants by transplacental antibody transfer is short, perhaps 6 months at most [5]. Interest in maternal aP immunization has grown because, despite decades of routine infant and childhood vaccination against the disease, pertussis cases began to increase in many countries between 2005 and 2010, with an unusual rise in deaths among young infants [6]. Where it is introduced, maternal immunization supplements routine infant vaccination, which can produce significant herd immunity at high coverage levels. For the whole-cell pertussis vaccine the infant target vaccination coverage necessary to begin to eliminate pertussis is thought to be 90%-95% [3].

To inform decisions about investing in maternal aP immunization, we compare two models, one static, one dynamic, that were developed for the same project and with the same purpose – to explore the public health impacts, costs, and cost-effectiveness of maternal immunization in low- and middle-income countries (LMICs) [7]. The static model, previously published, was parameterized separately for 3 example LMICs: Brazil, Nigeria, and Bangladesh [8]. The dynamic model, reported in this issue [9], was built with Brazilian data and then partially re-parameterized to evaluate maternal immunization for Nigeria and Bangladesh. Dynamic models require much more data – of good quality – and take longer to build, so it was not efficient to try to fit a separate model for each country. Instead our strategy was to fit a dynamic model to one country, in this case Brazil, an upper-middle-income country with rich surveillance, hospitalization, and mortality data going back decades, and then use it to project results for others as well.

The purpose of this paper is to take advantage of the two models, built to evaluate the same intervention, in order to explore when static models are adequate for good public health decisions and when the extra effort required by dynamic models is worthwhile. Because the focus is model comparison, not the cost-effectiveness of maternal immunization in different countries, we limited the comparisons to Brazil. We defined scenarios for model comparison that focused on two key differences between static and dynamic models, herd immunity and time horizon. We hypothesized that the cost-effectiveness of maternal immunization, although directed at a small portion of the population, depends on the level of routine infant vaccination and whether that level is above or below the target vaccination coverage at which the disease begins to be eliminated.

## 2 Methods

This section describes the models briefly with an emphasis on common features and key differences. We then describe the scenarios defined for the comparisons. A full description of the static model can be found in [8] and of the dynamic model in [9]. Diagrams of the models are included in Appendix A. Base-case parameter values for both models and data sources are shown in Table 1.

**Table 1:** Parameter values (calibration range) used for model comparisons.

Symbol	Parameter	Source	Dynamic model value	Static model value		
**Disease parameters**						

λ	force of infection	Calculated within the model from contact rates and transmission probabilities per contact.		None in model		
$c_{ij}$	contact rate	Polish POLYMOD matrix [Appendices 3 and 7 in 9; 10]	Adjusted for Brazilian/Polish household size and in the calibration process	None in model		
$q_{j}$	transmission probability per contact	[Appendix 7 in 9, 11]	The probabilities in Table A below were adjusted during the calibration process	None in model		
γ	1/infectious period	[12]	1/21 days	None in model		
$\rho_{1}$	reporting rate	Determined by calibration in dynamic model	infants: 8.3%, 1-9y: 7.1%, 10y+: 6.2%	10% for infants		
$\rho_{2}$	reporting rate among I_2_	Determined by calibration	40% of the primary case rates	None in model		
$\mu_{p}$	pertussis mortality, deaths per 1000 population by age	Brazilian Ministry of Health, National Mortality Information System (SIM). [13], [14]	Brazil, 1999–2016		2007	2014
				0–1 m	0.0187	0.1441
				2–3 m	0.0167	0.0965
				4–5 m	0.0021	0.0186
				6–8 m	0.0000	0.0042
				9–11 m	0.0000	0.0000
	all-cause mortality, deaths per 1000 population by age	Brazilian Ministry of Health, National Mortality Information System (SIM). [13], [14]	Brazil, 1999–2016	0–1 m	72.670	62.118
				2–3 m	8.437	6.872
				4–5 m	4.458	3.496
				6–8 m	3.262	2.281
				9–11 m	2.241	1.615
	pertussis hospitalizations, admissions per 1000 population by age	Brazilian Ministry of Health, National Public Health System (SUS) National Hospitalization Database (SIH-SUS) [15], [14].	Brazil, 1999–2016	0–1 m	0.694	4.334
				2–3 m	0.804	4.666
				4–5 m	0.290	1.891
				6–8 m	0.069	0.534
				9–11 m	0.033	0.204
	pertussis outpatient cases (all confirmed minus hospitalized confirmed), cases per 1000 population by age	Brazilian Ministry of Health, National Information System for Notifiable Diseases (Sinan) [16], [14]	Brazil, 1999–2016	0–1 m	0.0375	0.414
				2–3 m	0.0794	0.834
				4–5 m	0.0375	0.549
				6–8 m	0.0194	0.283
				9–11 m	0.0138	0.146
	population by age for probabilities	Brazilian Ministry of Health, Department of Informatics [17]	Brazil, 1999–2016	Brazil 2007 and 2014		
	DALY weight for pertussis disability	Global Burden of Disease (GBD) Collaborative Network [18]	0.051 (0.032–0.074)	None in model		

**Vaccine parameters(see note)**						

$\nu_{1}$	wP vaccine coverage (1st dose)	Derived from survey data modeled by Colin Sanderson using methods reported in [19]. See Methods.	Brazilian surveys for 1996 and 2007 used to model trend in vaccine coverage, 1999–2016. See Methods for projections.	See Methods and Appendix B.		
$\nu_{2}$	wP vaccine coverage (2nd dose)					
$\nu_{3}$	wP vaccine coverage (3rd dose)					
$\theta_{C}$	proportion of wP vaccine failure	[20]	0.1 (0–0.15)	0.1 (0–0.15)		
$\psi_{1}$	wP effectiveness (1st dose)	[21]	0.68 (95%CI: 0.456–0.811)	Same but doses 2 and 3 are combined: efficacy 95%		
$\psi_{2}$	wP effectiveness (2nd dose)	[21]	0.92 (95% CI: 0.847–0.957)			
$\psi_{3}$	wP effectiveness (3rd dose)	[21]	0.99 (95% CI: 0.989–1.000)			
$\nu_{M}$	maternal vaccine coverage	Brazilian Ministry of Health, National Immunization Program [22]	10.9% for 2014***48.4% for 2015***33.8%-70% for 2016–2030	70%		
$\theta_{M}$	proportion of maternal vaccine failure	[23]	0.1 (0–0.50)	0.1 (0–0.50)		
$\psi_{M}$	maternal vaccine effectiveness	[24]	0.91 (95% CI: 0.84–0.95)			
$\sigma_{V}$	waning rate	Determined by calibration	7.31 years (5–30)	Not in model		
$\sigma_{M}$	waning rate of maternal vaccine	[26]	3 months (2–6)			
$\sigma_{R}$	waning rate (natural infection)	[25]	20 years (10–50)			

**Cost parameters (2014 USD)**						

	maternal aP vaccine, dose	PAHO Revolving Fund Price List, 2014 [27]	9.55			
	infant wP vaccine, dose		2.71			
	wastage rate	WHO recommendation	5% (0–15%)			
	infant vaccine delivery cost	[28]	7.60			
	hospitalization cost – SURVIVOR, Brazil	Brazilian Ministry of Health, National Public Health System (SUS) National Hospitalization Database (SIH-SUS) [13]	0–1 m, 706.96			
			2–3 m, 543.74			
			4–5 m, 470.64			
			6–8 m, 492.15			
			9–11 m, 409.45			
			12–23 m, 490.94			
			2-4y, 413.29			
			5-9y, 406.25			
			10-19y, 392.96			
			20-39y, 392.93			
			40–64, 451.45			
			65+, 871.36			
	hospitalization cost – DIED Brazil	Brazilian Ministry of Health, National Public Health System (SUS) National Hospitalization Database (SIH-SUS) [13]	0–1 m, 1138.23			
			2–3 m, 1446.01			
			4–5 m, 1017.35			
			6–8 m, 910.62			
			9–11 m, 803.89			
			12–23 m, 519.28			
			2-4y, 519.28			
			5-9y, 519.28			
			10-19y, 519.28			
			20-39y, 519.28			
			40–64, 773.01			
			65+, 594.65			
	outpatient cost, per case (cases requiring outpatient care only)	Estimated from National Standardized pertussis treatment guidelines [29] using data from Brazilian Ministry of Health, Sources for Outpatient Costs [30]	< 4, 17.76			
			5–9, 14.16			
			10–19, 16.25			
			18+, 17.64			

Table A. Transmission probability per contact by age groups estimated at 1956 in England and Wales [11]

**Table:** 

Age groups	0y	1-2y	3-4y	5-9y	10-14y	15-24y	25-39y	40-59y	60+y

Transmission probability per contact	8.0 × 10^−4^	9.0 × 10^−4^	8.2 × 10^−4^	8.6 × 10^−4^	4.4 × 10^−4^	1.6 × 10^−4^	1.4 × 10^−4^	0.05 × 10^−4^	1.0 × 10^−4^

Note. Vaccine is assumed to be equally effective against infection and disease.

### 2.1 Static model

The static model, a decision tree built in TreeAge Pro (© TreeAge Software, Williamstown, Massachusetts), compares two strategies over an infant’s first year: (1) Maternal aP immunization plus routine infant whole-cell (wP) vaccination versus (2) Routine infant wP vaccination alone (Appendix A, Figure A-1). Maternal immunization plus routine infant vaccination branches according to whether or not the mother receives aP vaccine. After that, both strategies model the probability that an infant receives whole-cell-pertussis-containing (wP) vaccine during its first year in each of five age intervals, defined below. For this paper the static model in [8] was revised to include inpatient and outpatient treatment costs.

### 2.2 Dynamic model

Four variants of a compartmental, age-stratified, dynamic model that included the entire population, not just mothers and infants, were tested against monthly data for the years 1999–2016 [9]. (S = Susceptible, I = Infected/Infectious, R = Recovered.)

o SIR: Those who recover are immune for life.
o SIRS: Immunity wanes and repeat infections can occur.
o ${\mathrm{SIRS}}_{2}I_{2}$: Immunity wanes, individuals become susceptible again, and repeat infections can occur. Repeat infections are less serious so less likely to be reported.
o ${\mathrm{SIR}}_{2}^{B}I_{2}$: Immunity wanes and repeat infections are less serious so less likely to be reported. Those susceptible despite vaccination or previous infection are less susceptible. Exposure to infection can boost existing immunity.

Calibration was used to select some of the parameter values for each model variant. The calibration process selected parameter values that led to the best fit between the projections and the projection targets, incident pertussis cases and deaths observed over the period 1999–2016. For the calibration process model parameters were divided into two groups, those that were reasonably certain and those that were highly uncertain. The reasonably certain parameters were kept at their base-case values, which came from the published literature. The highly uncertain parameters were varied by means of 6 multipliers for contact rates between age groups, 3 for the reporting rate for outpatient cases, and 3 for the transmission probabilities per contact. To keep the computations manageable the multipliers were defined for 3 aggregated age groups (<1, 1–9, and 10 + years of age). The duration of immunity conferred by routine vaccination of infants with wP vaccine was also highly uncertain, so a total of 13 parameters were fitted by calibration. Akaike’s Information Criterion (AIC) was used to compare the fit of the four model variants. The third variant, ${\mathrm{SIRS}}_{2}I_{2}$, was the best-fitting model (Appendix A, Figure A-2), although the second,SIRS, was close and produced similar results. See [9] for more details about the dynamic model.

### 2.3 Common features of the models

#### 2.3.1 Strategies evaluated

Both models evaluate the cost-effectiveness of maternal aP immunization plus routine primary wP infant vaccination on the schedule used in Brazil (doses recommended at 2, 4, and 6 months), versus routine primary wP infant vaccination alone. Neither model includes the booster doses at 15 months and 4 years of age that are part of the recommended Brazilian schedule.

#### 2.3.2 Model outcomes

Both models project changes in deaths from pertussis, deaths from all causes, pertussis hospitalizations, pertussis outpatient cases, costs of maternal and infant pertussis vaccination, costs of pertussis outpatient and inpatient treatment, and cost-effectiveness under different scenarios.

#### 2.3.3 Data sources

Both models use national data for Brazil, as shown in Table 1.

#### 2.3.4 Age groups

Both models divide the first year of life into 5 age intervals – 0–1 month, 2–3, 4–5, 6–8, and 9–11 months – to capture the higher risks of pertussis at younger ages and to model the infant vaccination schedule in realistic detail. In each infant age interval, following receipt of the vaccine (or not) and development (or not) of protection derived from maternal aP or infant wP vaccination, the infant can die of pertussis, die of other causes, or survive.

#### 2.3.5 Costs

If the infant dies of pertussis, costs are incurred for treatment. If the infant survives, costs are incurred for subsequent doses of vaccine (if received), and, if the infant contracts pertussis, for pertussis treatment (hospital or outpatient). The same choices repeat at the next age interval. Vaccination is modeled by dose (1, 2, or 3) and an infant who does not receive a scheduled dose in one age interval is eligible to receive it in the next.

### 2.4 Key differences between the models

#### 2.4.1 Time horizon

The static model estimates annual outcomes for one year at a time based on data for that year. The dynamic model uses monthly data for 1999–2016 to project outcomes for 2017–2030, the base-case time horizon.

#### 2.4.2 Age groups included

The static model includes only pregnant women and infants (<1 year). The dynamic model includes the entire population, grouped by age as follows: the same five age groups < 1 year, 12–23 months, 2–4 years, and 5–9, 10–14, 15–19, 20–29, 30–39, 40–49, 50–59, 60–69, 70–79 and 80 + years.

#### 2.4.3 Measure of health outcome

The static model projects only years of life, and thus reports cost-effectiveness as the cost of maternal immunization plus infant vaccination, compared with infant vaccination alone, per year of life gained by maternal immunization. The dynamic model incorporates the disutility of the weeks that an infant who contracts pertussis is ill, using disability weights, and thus its cost-effectiveness ratios are the cost per disability-adjusted life-year (DALY) averted of maternal immunization plus infant vaccination, compared to infant vaccination alone. Since, however, the disutility associated with pertussis lasts only a few weeks, the primary health gain comes from lives saved, so the reader can safely interpret the cost-effectiveness ratios from the two models as comparable.

### 2.5 Scenarios defined for model comparisons

Two scenarios were defined to compare the models. Except as noted, the scenarios use the base-case parameter values.

*Scenario 1. Herd Immunity.* The first scenario evaluates maternal aP immunization against a range of coverage levels for infant vaccination. To represent that range we used infant vaccination coverage rates modeled from national household surveys by the methods in [19]. Two national surveys were available for Brazil, for 1996 and 2007. Since we wanted to explore the consequences not only of observed infant coverage levels in Brazil, but also of coverage levels outside that range, we made projections for five levels of infant coverage, two based on the Brazilian data and three based on data for three other LMICs. Brazil’s 2007 data came from a special national survey. The other four surveys were conducted under the auspices of the Demographic and Health Surveys (DHS): Brazil 1996, Nigeria 2008, India 2005 and Bangladesh 2011. Nigeria’s 2008 coverage is the lowest, Brazil’s 2007 coverage and India’s coverage are higher, but below the 90–95% target coverage range at one year of age. Brazil’s 1996 coverage is in the target range and Bangladesh’s survey reports rates above 95%. All five coverage levels are evaluated in the context of Brazil’s experience with pertussis and Brazil’s costs, so the cost-effectiveness results are interpreted as what would happen in Brazil at each coverage level.

Colin Sanderson modeled these national survey data to estimate the proportions of infants who received each of the three recommended doses of wP, by dose and week of age [19, 20a]. The vaccination probabilities used in the models – the probabilities that an infant receives dose 1, 2, or 3 in a given age interval – were derived from Sanderson’s modeled data using the 2-month spacing between doses in Brazil’s recommended schedule: dose 1 at 2 months, dose 2 at 4 months, and dose 3 at 6 months. To represent not just coverage but protection the calculations used data for the midpoint of each age interval since a few weeks must elapse after a dose is received before the infant has developed immunity. See Appendix B for details of the vaccination probabilities calculated for the models.

As infant vaccination coverage was varied, the coverage of maternal aP vaccine was held at 70% in a given year for the static model, and throughout the projection period, 2017–2030, for the dynamic model. This level of maternal coverage was judged to be reasonable by the Brazilian public health experts on the research team in light of the coverage already achieved in Brazil, first by tetanus-diphtheria (Td) immunization for pregnant women, and, since 2014, by TdaP.

The projections for Scenario 1 were run for two prices of maternal aP vaccine, the base case of $9.55/dose (the 2014 price available to Brazil through the Pan American Health Organization) and a low price of $1/dose, such as might be available to low-income countries. Since maternal immunization has been implemented in Brazil by replacing Td vaccination for pregnant women with TdaP, we assumed no additional delivery cost associated with aP immunization, and since the cost of the tetanus and diphtheria components is very small we treated the full vaccine price as the cost of aP.

For each level of infant coverage and maternal vaccine price the dynamic model estimated a single cost-effectiveness ratio for the projection period, 2017–2030. The static model estimated cost-effectiveness ratios for two years, 2007, an ordinary endemic year, and 2014, the peak of the pertussis resurgence in Brazil.

*Scenario 2. Time Horizon.* As is typical for dynamic models, the dynamic model estimates an average ratio that represents maternal immunization’s cost-effectiveness over the model’s time horizon for projections (2017–2030 for the base case). The static model estimates a cost-effectiveness ratio for a single year, based on the conditions for that year. To explore the effect of the differences in time horizon between the two models, we held infant vaccination coverage at the level of Brazil 2007, and maternal aP coverage at 70%, for all the projections for this scenario.

The dynamic model then projected cost per DALY averted for three time horizons: 2017–2025, 2017–2030, and 2017–2045. The static model projected cost-effectiveness for 2007 and 2014. A static model can, if populated with data averages, project average cost-effectiveness over multiple years. To explore the effect of doing this, the static model was also used to make projections based on the average probabilities of death from other causes, death from pertussis, pertussis hospitalization, and pertussis outpatient cases for the years 1999–2016, the same period on which the dynamic model was calibrated.

### 2.6 Uncertainty

We calculated 90% confidence intervals around the cost-effectiveness estimates of the static model using the probabilistic version of that model. As explained in [8] the probabilistic version assigns a distribution to each parameter and the model then estimates cost-effectiveness ratios repeatedly, each time using new sets of values randomly drawn from these distributions. Each 90% interval is based on 1000 such estimates and shows the lower and upper bounds that, between them, include 90% of the estimates. Following ISPOR-SMDM recommendations [2], the dynamic model was not made probabilistic and no confidence intervals are presented for it. Instead we note when its projections fall in the confidence intervals defined for the static model.

## 3 Results

This section presents cost effectiveness ratios for maternal immunization for each of the scenarios just described. Costs are in 2014 US dollars.

### 3.1 Scenario 1. Herd immunity

The first scenario evaluates the cost-effectiveness of maternal aP immunization at five different levels of routine infant wP coverage. For short, the five levels are referred to as *Low* (Nigeria 2008), *Moderate1* (Brazil 2007), *Moderate2* (India 2005), *High* (Brazil 1996), and *Highest* (Bangladesh 2011). The percentages of infants who had received at least one dose of pertussis vaccine by 26 and 52 weeks of age will help convey the differences among them: Nigeria (44%, 49%), Brazil 2007 (59%, 90%), India (69%, 72%), Brazil 1996 (89%, 94%), and Bangladesh (96%, 97%). The complete infant vaccination schedule, shown in Appendix B, is more complex and reflects doses delivered in each age interval during the first year.

#### 3.1.1 Static model

Maternal immunization saves infant lives, but at the price Brazil paid for maternal aP vaccine in 2014, $9.55/dose, the additional costs outweigh the savings in treatment from fewer cases of infant pertussis (Table 2). The additional cost per life-year increases as routine infant vaccination coverage rises from *Low* to *Highest*. At 2007 pertussis incidence, the cost rises from $12,295/life-year at *Low* coverage to $15,467/life-year at the *Highest* coverage, an increase of 26%. Cost per life-year is much lower at 2014 pertussis incidence, even at a maternal vaccine price of $9.55/dose, because rates of disease and death among infants were substantially higher in 2014, the peak of the resurgence, than in 2007 (see Table 1). In 2014 the additional cost of maternal immunization was $1,061/life-year at *Low* infant coverage and $1,468/life-year at the *Highest* infant coverage.

**Table 2:** Cost per life-year/DALY (2014$) by type of model, maternal vaccine price, infant vaccine coverage, and time period.

Infant vaccine coverage^a^	Static model									Dynamic model
	Cost/life-year	90% confidence interval^b^		Cost/life-year	90% confidence interval^b^		Cost/life-year	90% confidence interval^b^		Cost/DALY
	**2007**	10%	90%	**2014**	10%	90%	**1999**–**2016**	10%	90%	**2017**–**2030**
	Maternal vaccine price **$9.55/dose**									

Low	**$12,295**	$7,457	$35,506	**$1,061**	$544	$2,677	**$6,563**	$3,797	$18,338	**-$1,844**
Moderate1	**$12,469**	$6,031	$31,278	**$1,084**	$278	$2,039	**$6,658**	$3,322	$16,099	**$3,194**
Moderate2	**$13,140**	$6,751	$34,832	**$1,172**	$442	$2,448	**$7,025**	$3,502	$19,757	**$33,939**
High	**$14,861**	$7,364	$41,439	**$1,392**	$550	$2,956	**$7,962**	$4,106	$21,601	**$1,265,552**
Highest	**$15,467**	$9,650	$69,078	**$1,468**	$906	$3,788	**$8,290**	$5,353	$37,818	**$2,084,122**

	Maternal vaccine price **$1/dose**									

Low	**$444**	**−**$652	$2,680	**−$654**	**−**$951	**−**$362	**−$121**	**−**$850	$1,006	**−$2,672**
Moderate1	**$463**	**−**$952	$1,910	**−$649**	**−**$954	**−**$412	**−$110**	**−**$956	$703	**−$1,966**
Moderate2	**$535**	**−**$576	$2,306	**−$634**	**−**$957	**−**$384	**−$69**	**−**$775	$832	**$841**
High	**$721**	**−**$566	$2,858	**−$595**	**−**$916	**−**$334	**$35**	**−**$772	$1,121	**$124,425**
Highest	**$787**	**−**$153	$5,335	**−$581**	**−**$838	**−**$221	**$72**	**−**$663	$1,961	**$206,487**

Note. The static model estimates life-years, the dynamic model disability-adjusted life-years. See Methods.

a. See the first paragraph of the results section and Appendix B for detailed information about these coverage levels.

b. See Methods for an explanation of how the intervals were calculated.

At a maternal vaccine price of $1/dose, cost per life-year is substantially lower under both 2007 and 2014 pertussis incidence, even as it again rises with the level of routine infant vaccination coverage (Table 2). Under 2007 conditions, the cost is less than $1,000/life-year at all levels of infant vaccination coverage. Under 2014 conditions, maternal immunization is cost-saving at every level of infant coverage.

The static model was also used to project the cost-effectiveness of maternal immunization under average conditions for the period 1999–2016, a scenario more like that projected by the dynamic model. Under average conditions and a maternal vaccine price of $9.55/dose, the additional cost of maternal immunization is intermediate between the estimates for 2007 and 2014, rising from $6,563/life-year at *Low* infant coverage to $8,290 at the *Highest* coverage.

#### 3.1.2 Dynamic model

Like the static model, the dynamic model estimates that maternal immunization saves lives, usually at additional cost, although it can be cost-saving in some conditions, especially when the price of the maternal vaccine is very low.

**Fig. 1:**
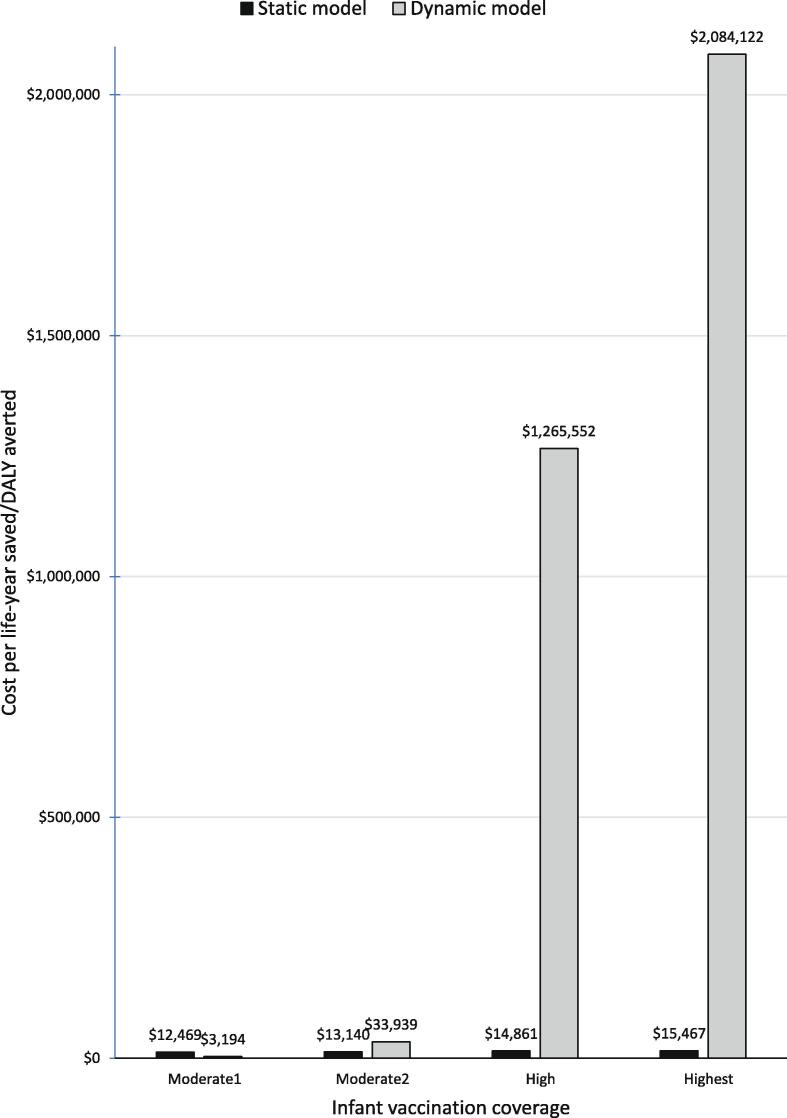
Effect of Herd Immunity: Cost per life-year saved/DALY averted by type of model, maternal vaccine price, $9.55/dose.

The pattern of cost/DALY projected by the dynamic model as routine infant vaccination coverage rises, however, stands in stark contrast to that of the static model. At both prices for maternal aP vaccine, maternal immunization is projected to be cost-saving at low infant vaccination coverage. But as infant coverage rises cost/DALY averted rises sharply. With infant coverage at the *High* and *Highest* levels, both of which are high enough to begin to eliminate the disease from the population, maternal aP immunization becomes a very expensive way to prevent infant disease and death: at the *Highest* level of infant coverage, and a maternal vaccine price of $9.55/dose, the cost is $2 million per DALY averted (Table 2 and Fig. 1). At the much lower price of $1/dose, it is $206,487/DALY (Table 2).

The costs per DALY (or life-year) estimated by the two models are not close. The estimates from the dynamic model fall outside the 90% interval for the static model’s estimates (Table 2; CIs are not included in Fig. 1 because they would not show up given the difference in cost-effectiveness ratios depicted there).

### 3.2 Scenario 2. Time Horizon: Average versus Single-Year estimates

The second scenario examines the impact of the different time horizons used for making estimates in the static and dynamic models. Table 3 shows estimates for three time periods for each model. All the estimates are based on infant vaccination coverage at the level of Brazil 2007 (*Moderate1*), for the period shown for the static model and for the entire projection period for the dynamic model, and a maternal vaccine price of $9.55/dose.

**Table 3:** Cost per life-year/DALY by type of model and time horizon.

Static model (90% confidence interval)			Dynamic model		
2007	2014	1999–2016	2017–2025	2017–2030	2017–2045
$12,469	$1,084	$6,658	$6,785	$3,194	$2,511
$6,021–30,397	$312–2,108	$3,251–16,791			

Note. The static model estimates life-years, the dynamic model disability-adjusted life-years.

#### 3.2.1 Static model

The static model’s estimates reflect pertussis incidence in the specified year – low incidence in 2007, high incidence in 2014, and in between those two extremes over the longer period, 1999–2016. Maternal aP immunization adds to costs in all three periods, but costs the most per life-year saved in 2007, the least in 2014, and, again, between those two extremes in 1999–2016. Note that Table 2 also showed the results for 1999–2016 at Brazil’s 2007 infant vaccination coverage (Moderate1). There they showed how the cost-effectiveness of maternal immunization changes as infant coverage changes. In Table 3 they emphasize the effect of looking at a time period longer than one year.

#### 3.2.2 Dynamic model

The dynamic model’s projections fall in the same general range as those from the static model. The dynamic model also shows, however, that cost per DALY averted declines as the time horizon of the projection grows longer. The reason for this pattern is that, at the infant coverage of Brazil 2007, the dynamic model projects disease under routine infant vaccination *alone* rising from 2017 to a peak at 2025, then declining slowly but remaining above the 2017 level through 2045. Under maternal immunization *plus* routine infant vaccination, the level of disease declines slowly and steadily from 2017. As a result, the longer time horizons involve higher average levels of disease under infant vaccination alone, maternal immunization prevents more disease, and the cost/DALY declines as the time horizon lengthens through 2045. Over still longer time horizons cost/DALY might remain steady or even begin to rise, depending on the level of infant coverage.

## 4 Discussion

Our models, built to evaluate maternal aP immunization, and using the same data sources for the same middle-income country, Brazil, make it possible to explore the well-known differences between static and dynamic models, not just conceptually but by comparing their estimates of the cost-effectiveness of maternal aP immunization.

The main finding of our analysis is that the background effect of routine infant pertussis vaccination, operating through herd immunity, is critical to the cost-effectiveness of maternal aP immunization. The infant vaccination coverage level required to eliminate pertussis is thought to be in the range 90–95% [3]. Coverage at the two highest levels used in our projections are at or above 90–95%. With coverage that high in the model, there is so little pertussis circulating in the population that newborns are at almost no risk, even without maternal immunization. The dynamic model makes this clear as its estimate of the cost of averting a DALY rises sharply when infant coverage is in this range, making maternal immunization very expensive. The static model fails to reflect the sharp rise in cost per life-year when pertussis drops to such low levels in the population and shows only a much more modest rise.

The dynamic model also shows maternal aP immunization to be cost-saving at low infant coverage, even when the static model does not. When infant coverage is low, the level of pertussis circulating in the population rises and feeds back into a higher risk for unvaccinated infants.

It is important to note that these conditions do not apply to all maternal immunization. Maternal immunization to protect infants against group B streptococcal (GBS) disease, for example, is a case in which only pregnant women are vaccinated – there is no vaccination of infants or others in the population [31]. In that case Brisson’s comment applies [1]: “If only a small proportion of the population is immunized (low coverage or targeted vaccination), or the vaccine does not prevent the circulation of the pathogen (as occurs with some vaccines), then herd-immunity effects are negligible. Under such conditions, static and dynamic models produce similar results.” The results of this analysis show that the effects of a targeted vaccination program are different when it takes place against a background of vaccination of the larger population. When an intervention such as maternal acellular pertussis immunization, which is too small to have significant herd immunity effects itself, takes place against a background of vaccination in the rest of the population, a dynamic model is crucial to accurate estimates of its cost-effectiveness.

Given recent modeling guidelines [2] and the trend in the literature toward dynamic transmission models, is there a role for static models when vaccination is widespread in the population? The rest of the discussion considers that question.

### 4.1 Advantages of dynamic models

The principal advantage of dynamic models is the purpose for which they were designed, the ability to simulate the effects of herd immunity and thus the effects of interventions against infectious disease in one group of the population on the rest of the population. As our results have shown, static models can be misleading when used to evaluate an intervention that, while itself targeted to a small proportion of the population, takes place against a background that includes vaccination of large groups of the population. The results of a static model are, of course, particularly misleading when background vaccination reaches the level at which herd immunity becomes a significant factor.

Because they project the pattern of disease incidence over time, dynamic transmission models can show the path disease incidence follows as an intervention is introduced and becomes widespread, while static models cannot. For a longer planning horizon the averages produced by dynamic models are thus appropriate. For a shorter horizon, e.g., during an epidemic, static models may be sufficient. Note that static models do not usually incorporate the long-term duration of immunity conferred by a vaccine, because their time horizons are short. The very short period of immunity conferred by maternal aP vaccine played a role in this static model, which focused on the first year of life, but the longer period of immunity conferred by infant vaccination did not.

Although not demonstrated in this paper, since dynamic models incorporate all groups in the population they can evaluate interventions in any group – adolescent boosters, for example – and their population effects. It also is worth noting that the direct impact of vaccination on pregnant women was not included in this dynamic model (nor in the static model).

### 4.2 Advantages of static models

The principal advantages of static models are that they can be built more quickly and are easier to use. That makes it possible to get answers to pressing policy questions more quickly. The static model reported here was complete and providing results a year after the project was funded, while the dynamic model took another year to reach that point. As a result, static models may serve for preliminary exploration of a situation involving an infectious disease, perhaps while a dynamic model is being developed. It is worth noting that the direction of change and the principle drivers of change were the same in the static and dynamic models presented here, although the magnitudes of change were often very different.

Static models require less data than dynamic models and little or no use of data that, if not actually unobservable, are unavailable, such as contact rates and probabilities of transmission of infection upon contact. In a dynamic model these parameters are often assigned values through calibration, the process in which a range of plausible values is tried and the values that produce the best fit between the model’s projections and the projection targets, such as disease incidence, are selected. The calibration process is useful in itself, providing information about the most likely values for these parameters, but is computationally intensive and time-consuming.

For the same reason, it is easier to change parameter values in a static model and to explore the impact of the change. Changing parameter values that were determined by calibration in a dynamic model can require re-calibrating the model.

Poor data limit both types of models. Static models can be tailored to the available data more easily than can dynamic models, which, by their nature, need to simulate the disease process. Static models can also more easily incorporate probabilistic sensitivity analysis [2]. While static models can thus more easily explore the implications of uncertainty about key parameters ([31] is an example), neither type of model can overcome the problems caused by seriously inaccurate data. In this project we found, for example, that administrative data on vaccine coverage overstated true coverage to such an extent as to show that pertussis should have been eliminated, leaving no role for maternal immunization; the problem was not the model’s estimates but the inaccuracy of the data, and was resolved in this case by locating more accurate coverage data from household surveys [19].

### 4.3 Conclusion

Because of the greater ease with which they can be built and used, static models may continue to play a useful role in the evaluation of interventions against infectious diseases, especially interventions that are small relative to the disease and that do not occur against a background of other interventions that alter disease transmission in the population. They can be particularly useful in providing policymakers with quick and early insights during the development of the investment case for an intervention. When, however, the intervention being evaluated will be applied in a situation where other interventions against the same disease, such as infant vaccination, are widespread enough to produce herd immunity, dynamic models are needed to produce accurate estimates of the target intervention’s health outcomes, costs, and cost-effectiveness. Our results, together with the companion papers in this issue by Kim [9] and Luz [32] support the recommendation that dynamic models are needed to evaluate the cost-effectiveness of interventions that are implemented in these conditions in LMICs.

## Funding

10.13039/100000865Bill & Melinda Gates Foundation Grant OPP1124529

## Declaration of Competing Interest

The authors declare the following financial interests/personal relationships which may be considered as potential competing interests: Dr. Sinha was at Rutgers School of Public Health until August 2016, but is currently employed by Merck Research Laboratory, a division of Merck & Co. Dr. Andrade has received lecture fees and travel grants from GlaxoSmithKline and Pfizer. Dr. Minamisava has received travel grants from GlaxoSmithKline. The other authors have no conflicts of interest.
