# Supplementary material for: Comparison of static and dynamic models of maternal immunization to prevent infant pertussis in Brazil
Source: Vaccine. 2021 Jan 3;39(1):158–66. doi: 10.1016/j.vaccine.2020.09.006 (PMC7735374; doi:10.1016/j.vaccine.2020.09.006)
Supplement: Supplementary data 1 [file mmc1.docx]

Appendix A. Diagrams of the Models

Figure A-1. Schematic of the static model

Figure A-2. Schematic of the dynamic model (SIRS_2_I_2_)

**
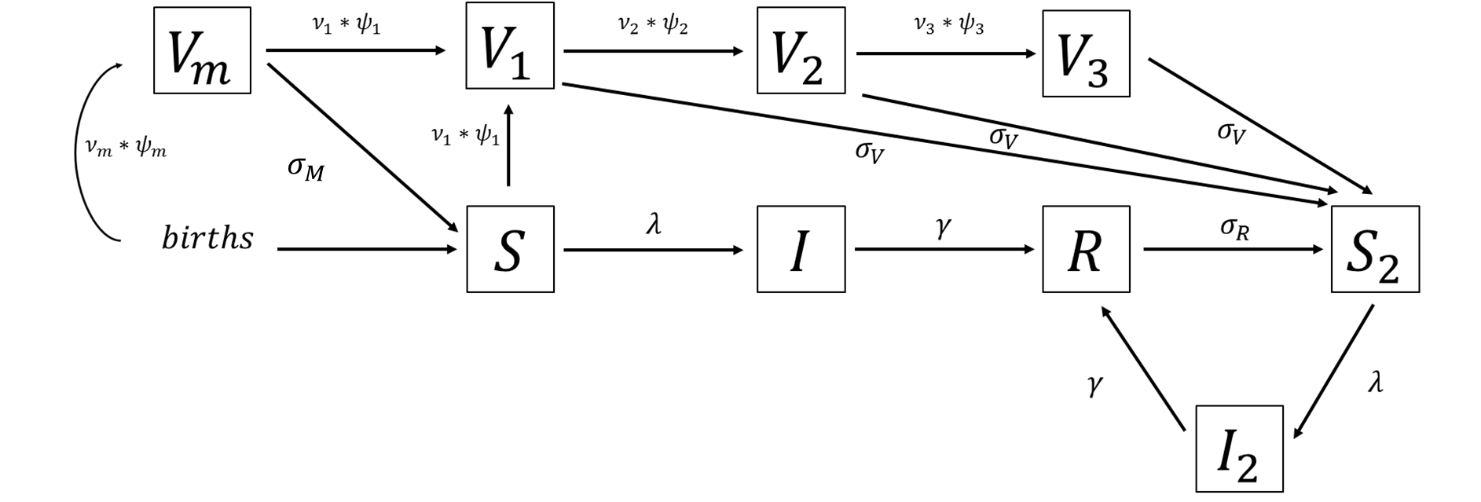
**

The model assumes that both vaccine-induced and naturally-acquired immunity wane and repeat infection has a lower reporting rate than primary infection. Brief descriptions of each symbol in the schematic is as follows:

S: Susceptible population

I: Infected and infectious population

R: Recovered and immune population from the infection

S_2_: Population with waning immunity from V or R compartments

I_2_: Population with secondary infection from S_2_

V_m_: Effectively immunized by maternal vaccination

V_1_: Effectively immunized by the 1st dose of child vaccination

V_2_: Effectively immunized by the 2nd dose of child vaccination

V_3_: Effectively immunized by the 3rd dose of child vaccination

$\psi_{i}$: Proportion moving to protected compartments after vaccination (considering both primary vaccine failure and vaccine efficacy)

$v_{i}$: Proportion of individuals to be vaccinated (vaccine coverage)

$\sigma_{V}$: Waning rate of wP vaccine-induced immunity

σ_M_: Waning rate of aP vaccine-induced immunity

$\sigma_{R}$: Waning rate of natural infection-induced immunity

$\lambda$: Force of infection

$\gamma$: Recovery rate

Appendix B. Calculation of the Infant Vaccine Coverage Probabilities

Using individual data on applied vaccine doses by children from these two surveys, Colin Sanderson provided us with vaccination coverage modeled by dose and week of age up to 3 years of age for all five vaccination coverage surveys used in this analysis. Coverage for each dose was cumulative so that the proportion of infants vaccinated rose monotonically with week of age. The methods are described in Clark A, Sanderson C. Timing of children’s vaccinations in 45 low-income and middle-income countries: an analysis of survey data. *Lancet* 2009; 373:1543–9. Data for four of the countries (Brazil 1996, Bangladesh, India, and Nigeria) were prepared for that articles. Sanderson applied the same methods to the 2007 Brazilian survey data, which were provided by the survey’s principal investigator Dr José Cassio de Moraes.

The table below shows Sanderson’s data for weeks 26 and 52 in order to give a sense not only for the data but also for the differences in coverage across the five countries used in the analysis.

| Proportion of infants who had received pertussis vaccine (dose) at (week) | | | | | | |
| --- | --- | --- | --- | --- | --- | --- |
| Dose | Week | Nigeria (Low) | Brazil 2007 (Moderate1) | India (Moderate2) | Brazil 1996 (High) | Bangladesh (Highest) |
| 1 | 26 | 0.44 | 0.59 | 0.69 | 0.89 | 0.96 |
|  | 52 | 0.49 | 0.90 | 0.72 | 0.94 | 0.97 |
|  |  |  |  |  |  |  |
| 2 | 26 | 0.35 | 0.32 | 0.57 | 0.72 | 0.91 |
|  | 52 | 0.41 | 0.79 | 0.64 | 0.88 | 0.95 |
|  |  |  |  |  |  |  |
| 3 | 26 | 0.24 | 0.09 | 0.40 | 0.03 | 0.77 |
|  | 52 | 0.33 | 0.61 | 0.53 | 0.74 | 0.91 |

From Sanderson’s modeled data we calculated the probability that an infant in each of five age intervals (0-1, 2-3, 4-5, 6-8, and 9-11 months) received a given dose. To represent not just coverage but protection the calculations used data for the midpoint of each age interval since a few weeks must elapse after a dose is given before the infant has developed immunity. The calculation of the probabilities is explained in general in the paragraphs below and for each probability in the tables that follow.

For the first dose, the numerator of the probability was the proportion of infants who received a first dose between the midpoint of the previous age interval and the midpoint of the age interval of interest. For example, for the age interval 4-5 months the numerator was the proportion of infants who received the first dose at the midpoint of the 4-5-month age interval, minus those who had received it at the midpoint of the 2-3-month age interval. The denominator was the proportion of infants who had not received a first dose as of the midpoint of the previous age interval. The denominator for the example was thus 1 minus the proportion of infants who had received the first dose at the midpoint of the 2-3-month age interval.

For doses 2 and 3 the numerator of the probability was the proportion of infants who received that dose between the midpoint of the previous age interval and the midpoint of the age interval of interest. The denominator was defined to take into account that the infant must have received dose 1 to be eligible for dose 2, or dose 2 to be eligible for dose 3, and, applying the Brazilian schedule for infant vaccination to all five surveys, must have received that dose 2 months or more before the dose of interest. For the age intervals 2-3 months and 4-5 months, each exactly two months long, the denominator was thus the proportion of infants who had received the required dose, say dose 1, at the midpoint of the previous age interval minus those who had already received the dose of interest, say dose 2, at the same time point; the difference was those who still needed dose 2 and were eligible to receive it. For the age intervals 6-8 and 9-11 months, each longer than two months, the denominators were defined as the proportion of infants who had received the preceding dose two months before the midpoint of the age interval of interest.

To obtain standard errors for the probabilities, we used the formula: SQRT((p*(1-p))/N) where p=proportion who received a given dose in a given age interval and N=number of infants in the survey.

The tables of probabilities used for the analysis follow.

| **NIGERIA, 2008 (Low)** | | |
| --- | --- | --- |
| p_dpt1_23m | 0.340000 | infants w DPT1 by 12 wks |
| p_dpt1_45m | 0.106061 | infants w DPT1 by 21 wks minus 12 wks/ 1 minus DPT1 by 12 wks |
| p_dpt1_68m | 0.067797 | infants w DPT1 by 32 wks minus 21 wks/ 1 minus DPT1 by 21 wks |
| p_dpt1_911m | 0.054545 | infants w DPT1 by 45 wks minus 32 wks/ 1 minus DPT1 by 32 wks |
| p_dpt2_45m | 0.941176 | infants w DPT2 by 21 wks/infants w DPT1 by 12 wks (2m earlier) |
| p_dpt2_68m | 0.454545 | infants w DPT2 by 32 wks minus by 21 wks/infants w DPT1 by 23 wks minus DPT2 by 21 wks |
| p_dpt2_911m | 0.333333 | infants w DPT2 by 45 wks minus by 32/infants w DPT1 by 36 wks minus DPT2 by 32 wks |
| p_dpt3_68m | 0.818182 | infants w DPT3 by 32 wks/infants w DPT2 by 23 wks (2m earlier) |
| p_dpt3_911m | 0.454545 | infants w DPT3 by 45 wks minus by 32 wks/infants w DPT2 by 36 wks minus DPT3 by 32 wks |

| **BRAZIL, 2007 (Moderate1)** | | |
| --- | --- | --- |
| p_dpt1_23m | 0.316770 | infants w DPT1 by 12 wks |
| p_dpt1_45m | 0.273029 | infants w DPT1 by 21 wks minus 12 wks/ 1 minus DPT1 by 12 wks |
| p_dpt1_68m | 0.376524 | infants w DPT1 by 32 wks minus 21 wks/ 1 minus DPT1 by 21 wks |
| p_dpt1_911m | 0.514348 | infants w DPT1 by 45 wks minus 32 wks/ 1 minus DPT1 by 32 wks |
| p_dpt2_45m | 0.715383 | infants w DPT2 by 21 wks/infants w DPT1 by 12 wks (2m earlier) |
| p_dpt2_68m | 0.765243 | infants w DPT2 by 32 wks minus by 21 wks/infants w DPT1 by 21 wks minus DPT2 by 21 wks |
| p_dpt2_911m | 0.834359 | infants w DPT2 by 45 wks minus by 32/infants w DPT1 by 36 wks minus DPT2 by 32 wks |
| p_dpt3_68m | 0.743789 | infants w DPT3 by 32 wks/infants w DPT2 by 21 wks |
| p_dpt3_911m | 0.734211 | infants w DPT3 by 45 wks minus by 32 wks/infants w DPT2 by 36 wks minus DPT3 by 32 wks |

| **INDIA, 2005 (Moderate2)** | | |
| --- | --- | --- |
| p_dpt1_23m | 0.530000 | infants w DPT1 by 12 wks |
| p_dpt1_45m | 0.297872 | infants w DPT1 by 21 wks minus 12 wks/ 1 minus DPT1 by 12 wks |
| p_dpt1_68m | 0.090909 | infants w DPT1 by 32 wks minus 21 wks/ 1 minus DPT1 by 21 wks |
| p_dpt1_911m | 0.066667 | infants w DPT1 by 45 wks minus 32 wks/ 1 minus DPT1 by 32 wks |
| p_dpt2_45m | 0.962264 | infants w DPT2 by 21 wks/infants w DPT1 by 12 wks (2m earlier) |
| p_dpt2_68m | 0.588235 | infants w DPT2 by 32 wks minus by 21 wks/infants w DPT1 by 23 wks minus DPT2 by 21 wks |
| p_dpt2_911m | 0.200000 | infants w DPT2 by 45 wks minus by 32/infants w DPT1 by 36 wks minus DPT2 by 32 wks |
| p_dpt3_68m | 0.851852 | infants w DPT3 by 32 wks/infants w DPT2 by 23 wks (2m earlier) |
| p_dpt3_911m | 0.312500 | infants w DPT3 by 45 wks minus by 32 wks/infants w DPT2 by 36 wks minus DPT3 by 32 wks |

| **BRAZIL, 1996 (High)** | | |
| --- | --- | --- |
| p_dpt1_23m | 0.699588 | infants w DPT1 by 12 wks |
| p_dpt1_45m | 0.563578 | infants w DPT1 by 21 wks minus 12 wks/ 1 minus DPT1 by 12 wks |
| p_dpt1_68m | 0.298626 | infants w DPT1 by 32 wks minus 21 wks/ 1 minus DPT1 by 21 wks |
| p_dpt1_911m | 0.220444 | infants w DPT1 by 45 wks minus 32 wks/ 1 minus DPT1 by 32 wks |
| p_dpt2_45m | 0.810366 | infants w DPT2 by 21 wks/infants w DPT1 by 12 wks (2m earlier) |
| p_dpt2_68m | 0.778305 | infants w DPT2 by 32 wks minus by 21 wks/infants w DPT1 by 21 wks minus DPT2 by 21 wks |
| p_dpt2_911m | 0.529659 | infants w DPT2 by 45 wks minus by 32/infants w DPT1 by 36 wks minus DPT2 by 32 wks |
| p_dpt3_68m | 0.950715 | infants w DPT3 by 32 wks/infants w DPT2 by 21 wks |
| p_dpt3_911m | 0.611181 | infants w DPT3 by 45 wks minus by 32 wks/infants w DPT2 by 36 wks minus DPT3 by 32 wks |

| **BANGLADESH, 2011 (Higfhest)** | | |
| --- | --- | --- |
| p_dpt1_23m | 0.810000 | infants w DPT1 by 12 wks |
| p_dpt1_45m | 0.736842 | infants w DPT1 by 21 wks minus 12 wks/ 1 minus DPT1 by 12 wks |
| p_dpt1_68m | 0.400000 | infants w DPT1 by 32 wks minus 21 wks/ 1 minus DPT1 by 21 wks |
| p_dpt1_911m | 0.000000 | infants w DPT1 by 45 wks minus 32 wks/ 1 minus DPT1 by 32 wks |
| p_dpt2_45m | 0.977273 | infants w DPT2 by 21 wks/infants w DPT1 by 14 wks (approx 2m earlier) |
| p_dpt2_68m | 0.700000 | infants w DPT2 by 32 wks minus by 21 wks/infants w DPT1 by 23 wks minus DPT2 by 21 wks |
| p_dpt2_911m | 0.250000 | infants w DPT2 by 45 wks minus by 32/infants w DPT1 by 36 wks minus DPT2 by 32 wks |
| p_dpt3_68m | 0.965909 | infants w DPT3 by 32 wks/infants w DPT2 by 23 wks (2m earlier) |
| p_dpt3_911m | 0.666667 | infants w DPT3 by 45 wks minus by 32 wks/infants w DPT2 by 36 wks minus DPT3 by 32 wks |
