# Supplementary material for: Comparison of static and dynamic models of maternal immunization to prevent infant pertussis in Brazil
Source: Vaccine. 2021 Jan 3;39(1):158–66. doi: 10.1016/j.vaccine.2020.09.006 (PMC7735374; doi:10.1016/j.vaccine.2020.09.006)
Supplement: Supplementary data 3 [file mmc3.pdf]

Clinical Infectious Diseases
SUPPLEMENT ARTICLE

What Pertussis Mortality Rates Make Maternal Acellular Pertussis Immunization Cost-Effective in Low- and Middle-Income Countries? A Decision Analysis

Louise B. Russell,[1] Sri Ram Pentakota,[2] Cristiana Maria Toscano,[3] Ben Cosgriff,[4] and Anushua Sinha[5]

[1]Department of Economics and Institute for Health, New Brunswick, and [2]Department of Surgery, New Jersey Medical School, Rutgers University, Newark; [3]Department of Community Health, Federal University of Goiás, Brazil; [4]Consultant, Westfield, and [5]Department of Health Systems and Policy, School of Public Health, Rutgers University, Piscataway, New Jersey

Background. Despite longstanding infant vaccination programs in low- and middle-income countries (LMICs), pertussis continues to cause deaths in the youngest infants. A maternal monovalent acellular pertussis (aP) vaccine, in development, could prevent many of these deaths. We estimated infant pertussis mortality rates at which maternal vaccination would be a cost-effective use of public health resources in LMICs.

Methods. We developed a decision model to evaluate the cost-effectiveness of maternal aP immunization plus routine infant vaccination vs routine infant vaccination alone in Bangladesh, Nigeria, and Brazil. For a range of maternal aP vaccine prices, one-way sensitivity analyses identified the infant pertussis mortality rates required to make maternal immunization cost-effective by alternative benchmarks ($100, 0.5 gross domestic product [GDP] per capita, and GDP per capita per disability-adjusted life-year [DALY]). Probabilistic sensitivity analysis provided uncertainty intervals for these mortality rates.

Results. Infant pertussis mortality rates necessary to make maternal aP immunization cost-effective exceed the rates suggested by current evidence except at low vaccine prices and/or cost-effectiveness benchmarks at the high end of those considered in this report. For example, at a vaccine price of $0.50/dose, pertussis mortality would need to be 0.051 per 1000 infants in Bangladesh, and 0.018 per 1000 in Nigeria, to cost 0.5 per capita GDP per DALY. In Brazil, a middle-income country, at a vaccine price of $4/dose, infant pertussis mortality would need to be 0.043 per 1000 to cost 0.5 per capita GDP per DALY.

Conclusions. For commonly used cost-effectiveness benchmarks, maternal aP immunization would be cost-effective in many LMICs only if the vaccine were offered at less than $1–$2/dose.

Keywords. pertussis; maternal immunization; mortality; cost-effectiveness; decision analysis.

One target of the United Nations Sustainable Development Goal 3 is to end preventable deaths of infants and children <5 years of age in low- and middle-income countries (LMICs) [1]. Despite widespread infant vaccination, pertussis can be fatal to very young infants before they are vaccinated, and may be resurging in some settings. Single-dose maternal acellular pertussis (aP) immunization during pregnancy, which confers immunity on infants through transplacental antibody transfer and reduces their exposure to pertussis by protecting their mothers, could prevent many of these deaths. Some high- and upper-middle-income countries have already added maternal aP vaccination to their adult immunization schedules [2–5].

The issue before LMIC governments and international funders is whether maternal aP immunization is a worthwhile use of public health funds, given competing public health priorities in these countries. Despite the possible resurgence, which may be a transient result of older, less effective infant vaccines and incomplete coverage, recent studies suggest that pertussis mortality among LMIC infants may be very low [6]. Information about pertussis mortality in LMICs is sparse and highly uncertain, but to decide whether maternal aP immunization deserves priority, governments and funders need to know whether enough deaths could be prevented to make it a cost-effective investment.

To address this issue, we developed a decision model to show under what conditions maternal aP immunization would be a good public health investment in LMICs. We used the model to identify the pertussis mortality rates (termed "mortality thresholds") at which maternal aP immunization would be considered cost-effective by several alternative cost-effectiveness benchmarks.

METHODS

The decision tree, built in TreeAge Pro (Williamstown, Massachusetts), compares 2 strategies over an infant's first year: (1) maternal immunization plus routine infant vaccination and (2) routine infant vaccination alone. Maternal immunization plus routine infant vaccination branches according to whether or not the mother receives aP vaccine. After that, both strategies

Correspondence: L. B. Russell, Institute for Health, Rutgers University, 112 Paterson St, New Brunswick, NJ 08901 (lrussell@ifh.rutgers.edu).

Clinical Infectious Diseases® 2016;63(S4):S227–35
© The Author 2016. Published by Oxford University Press for the Infectious Diseases Society of America. This is an Open Access article distributed under the terms of the Creative Commons Attribution License (http://creativecommons.org/licenses/by/4.0/), which permits unrestricted reuse, distribution, and reproduction in any medium, provided the original work is properly cited. DOI: 10.1093/cid/ciw558

Downloaded from http://cid.oxfordjournals.org/ by guest on November 12, 2016

model the probability that the infant receives routine diphtheria-tetanus-pertussis (DTP) vaccine. The first year is divided into 5 age intervals: 0–1, 2–3, 4–5, 6–8, and 9–11 months. In each age interval, following receipt (or not) of protection from maternal or routine infant vaccination, the infant can die of pertussis, die of other causes, or survive. If the infant survives, the same choices repeat at the next age interval. Vaccination is modeled by DTP dose so that an infant who does not receive a scheduled dose in one age interval is eligible to receive it in the next. Figure 1 shows a representative portion of the model, the first 2 age intervals for the arm on which pregnant women receive aP vaccine.

The focus of this analysis was the identification of infant pertussis mortality rates at which selected cost-effectiveness benchmarks would be achieved. We present results, from a healthcare system perspective, for 2 low-income countries, Bangladesh and Nigeria, and 1 middle-income country, Brazil, and for a range of maternal aP vaccine prices appropriate to each country. Table 1 summarizes the parameter values for each country, which are briefly described below. The Supplementary Technical Appendix provides more complete detail.

Vaccine Coverage

Maternal aP vaccine, which is offered late in pregnancy, could be provided through antenatal care or through programs that already provide pregnant women with tetanus toxoid. In LMICs, many pregnant women first attend antenatal care later in pregnancy and have only 1 visit before delivery. Thus, ANC1, the percentage of pregnant women with at least 1 antenatal visit, available from Demographic and Health Surveys (DHS), is a reasonable proxy for coverage in Bangladesh [14] and Nigeria [15]. For Brazil, which began offering maternal aP immunization in late 2014, we used unpublished data on maternal tetanus-diphtheria/tetanus-diphtheria-acellular pertussis (Td/Tdap) coverage in 2015, provided by the Surveillance Secretariat, Brazilian Ministry of Health.

To represent routine infant vaccination in Bangladesh and Nigeria, we used unpublished proportions of infants who received DTP1, DTP2, and DTP3, modeled by week of age from DHS data by Colin Sanderson of the London School of Hygiene and Tropical Medicine, based on the 2011 DHS for Bangladesh and the 2008 DHS for Nigeria; the modeling is similar to that in [24] but the data are more recent. Brazilian vaccination rates were estimated from unpublished data provided by the Goiania Municipal Health Department on doses of vaccine delivered to infants in Goiania municipality, by age. In the model, vaccination represents *protection* against pertussis, so we used proportions vaccinated at the midpoint of each age interval to represent infants who had not only received a dose but developed immunity from it. The numerator for each proportion was infants who received the specified dose in that age interval; the denominator was infants in the age interval who had not received the specified dose at an earlier age and thus were still eligible to receive it. The vaccination schedule, the standard 2/4/6-months schedule used in Brazil or the 6/10/14-weeks accelerated schedule used in Bangladesh and Nigeria [25], is naturally reflected in the proportions of children who have received DTP by the midpoint of an age interval.

Vaccine Efficacy

An English study found that maternal immunization based on a 3-component aP vaccine reduced pertussis cases in infants aged <3 months by 91% (95% confidence interval, 84%–95%) [4]. A single-component aP vaccine, currently being developed to reduce vaccine cost, may plausibly be somewhat less effective, although clinical trial data are not yet available. We therefore used a vaccine efficacy of 85% for our analyses. Passive immunity is stable for the first 3 months of life, but largely gone by 6 months [26], so in the model maternal aP immunization reduces infant deaths, in infants who have not yet received DTP shots of their own, only for the first 3 months. The efficacy of routine infant vaccine is from a German study [13].

Health Outcomes

During each age interval, the infant may die of pertussis, die of other causes, or survive to live an average life expectancy.

Downloaded from http://cid.oxfordjournals.org/ by guest on November 12, 2016

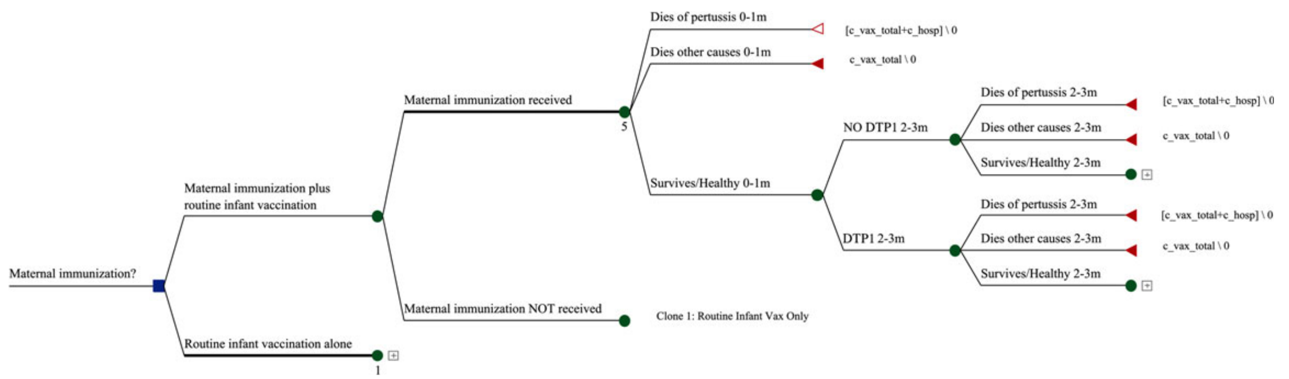

Figure 1. First 2 age intervals in the decision tree, maternal immunization branch. Abbreviation: DTP1, vaccine that is effective against diphtheria, tetanus, and pertussis.

Table 1. Key Model Parameters and Background Data by Country

Parameter	Bangladesh	Nigeria	Brazil	Distribution
Demographics				
Live births, No. (latest year available) [7]	2 933 000 (2012)	1 807 025 (2007)	2 832 590 (2013)	NA
Infant mortality rate, deaths/1000 live births, 2014 [8]	32.1	71.5	14.4	NA
Neonatal mortality rate, deaths/1000 live births, 2014 [8]	24.2	35	9.6	NA
Life expectancy at birth, y (range) [9]	71.0 (69.0–72.9)	52.3 (50.2–53.7)	74.1 (72.6–75.4)	Uniform
Discounted life expectancy at birth, 3% discount rate (range) [10]	27.82 (27.3–28.3)	22.94 (22.2–23.4)	28.47 (28.2–28.7)	Uniform
Discounted life expectancy at birth, 5% discount rate (range) [10]	18.39 (18.1–18.6)	15.78 (15.3–16.1)	18.75 (18.6–18.9)	Uniform
GDP per capita, 2014 [11]	$1086.80	$3203.30	$11384.40	NA
Pertussis mortality				
Probability of death from pertussis, first year of life (see Methods)		Ranged to determine mortality thresholds		Uniform
Pertussis deaths by age in first year, %, 2014 [12]				
0–1 mo		62.50		Constant
2–3 mo		29.17		Constant
4–5 mo		6.25		Constant
6–8 mo		1.04		Constant
9–11 mo		1.04		Constant
Probability of death from other causes [8]				
0–1 mo	0.02492	0.03838	0.01004	Constant
2–3 mo	0.00148	0.00700	0.00088	Constant
4–5 mo	0.00148	0.00700	0.00088	Constant
6–8 mo	0.00222	0.01048	0.00132	Constant
9–11 mo	0.00222	0.01048	0.00132	Constant
DTP vaccination by age and dose (see Methods)				
Probability of DTP1, if not received earlier				
2–3 mo (SE)	0.810 (0.010)	0.340 (0.006)	0.940 (0.002)	Beta
4–5 mo (SE)	0.737 (0.011)	0.106 (0.004)	0.360 (0.004)	Beta
6–8 mo (SE)	0.400 (0.012)	0.068 (0.003)	0.160 (0.003)	Beta
9–11 mo (SE)	0.200 (0.010)	0.055 (0.003)	0.040 (0.002)	Beta
Probability of DTP2, once DTP1 has been received				
4–5 mo (SE)	0.925 (0.007)	0.821 (0.005)	0.920 (0.002)	Beta
6–8 mo (SE)	0.636 (0.012)	0.417 (0.006)	0.790 (0.003)	Beta
9–11 mo (SE)	0.200 (0.010)	0.300 (0.006)	0.410 (0.004)	Beta
Probability of DTP3, once DTP2 has been received				
6–8 mo (SE)	0.924 (0.007)	0.750 (0.006)	0.940 (0.002)	Beta
9–11 mo (SE)	0.667 (0.012)	0.417 (0.006)	0.650 (0.004)	Beta
Infant vaccine efficacy [13]				
Efficacy in infants who received only 1 dose of wP vaccine (SE)		0.68 (0.09)		Beta
Efficacy in infants who received 2 or 3 doses of wP vaccine (SE)		0.95 (0.02)		Beta
Maternal vaccine efficacy (SE) [4]		0.85 (0.03)		Beta
Maternal vaccine coverage				
At least 1 antenatal care visit, % (SE; year) [14, 15]	78.6 (0.59; 2014)	65.8 (0.33; 2013)	NA	Beta
Coverage of maternal Td/Tdap, Brazil, % (range), 2015 (see Methods)	NA	NA	53.03 (40.3–60.2)	Beta
Maternal vaccine program costs				
Td plus monovalent aP vaccine, per dose, range (see Methods)	$0.50–$5.00	$0.50–$5.00	$4.00–$12.00	Uniform
Incremental vaccine delivery cost per dose (see Methods)		$0		NA
EPI vaccine infant program costs				
Pentavalent DTwP-HepB-Hib, per dose, 2016 (range) [16–19]	$2.23 ($1.40–$2.81)	$2.23 ($1.40–$2.81)	$2.30 ($2.19–$2.42)	Uniform
Incremental delivery cost per dose (range) [20–22]	$0.74 ($0.67–$0.81)	$5.83 ($5.25–$6.41)	$5.97 ($5.37–$6.57)	Uniform
Disease management costs				
Inpatient care, per day [23]	$5.70	$25.83	NA	NA
Length of hospital stay, d (see Methods)	6.0–9.2		NA	
Total cost (range)	$43.30 ($34–$52)	$196.32 ($155–$238)	NA	Uniform
Hospital cost for infants who died of pertussis, Brazil (SE) [12]	NA	NA	$1124 ($185)	Gamma

Source in brackets. All costs are in 2014 US dollars.

Abbreviations: aP, acellular pertussis; DTP, diphtheria-tetanus-pertussis; DTwP, diphtheria-tetanus-whole cell pertussis; EPI, Expanded Programme on Immunization; GDP, gross domestic product; HepB, hepatitis B; Hib, *Haemophilus influenzae* type b; NA, not applicable; SE, standard error; Td, tetanus-diphtheria; Tdap, tetanus-diphtheria-acellular pertussis; wP, whole-cell pertussis.

Downloaded from http://cid.oxfordjournals.org/ by guest on November 12, 2016

Survive/healthy includes infants who contracted pertussis (or other diseases) but recovered. The probability of each outcome depends on the infant's age and doses of DTP received.

To serve the purpose of the analysis—to identify infant pertussis mortality rates that make maternal aP immunization cost-effective—the model calculates pertussis deaths in 3 steps.

First, the overall probability of death from pertussis during the first year is set at any desired level. Second, the selected probability of pertussis death is distributed over the age intervals in the model using 2014 Brazilian data on pertussis mortality by age in hospitalized infants [12], which show that the majority of deaths occur in the first month and >90% in the first 2 months (Table 1). Third, to account for vaccination status, the model applies equations that express the pertussis death rate in an age interval as a weighted average of death rates by vaccination status—no DTP, 1 dose, ≥2 doses. When populated with data on vaccination status (the unpublished data from Colin Sanderson and the Goiania Municipal Health Department, noted above) and vaccine effectiveness [13], these equations yield probabilities by vaccination status within each age interval, thus completing the distribution of pertussis deaths by age and vaccination status. The Supplementary Technical Appendix provides the equations and example calculations.

Deaths from other causes were calculated using the United Nations Inter-agency Group's child mortality estimates for neonates and infants [8]. Neonatal mortality was subtracted from infant mortality, and postneonatal mortality was then distributed evenly over months 2–11. Mortality in the age interval 0–1 month is neonatal mortality plus, for the second month, the average monthly postneonatal mortality. Mortality for the remaining age intervals is average monthly postneonatal mortality for the appropriate number of months. Life expectancy (2010–2015) for surviving infants is also from the United Nations [9]; the range was obtained from data for the periods 2005–2010 and 2015–2020. To derive cost-effectiveness ratios, life expectancy was discounted at 3%/year using [10].

Costs

Costs were adjusted to 2014 US dollars using the World Bank's annual gross domestic product (GDP) deflator series [27] and average annual currency exchange rates [28]. All costs occur during the first year of life, so are not discounted.

The price of the maternal aP vaccine in development, which will contain tetanus, diphtheria, and a single pertussis antigen, is not known. We evaluated a range of plausible prices: $0.50–$5.00/dose for Bangladesh and Nigeria, and $4–$12/dose for Brazil, which currently pays $11.50/dose for a multivalent aP vaccine formulation [16]. The older antigens, tetanus and diphtheria, will likely cost so little that we treat total vaccine price per dose as the incremental cost of maternal aP. Because Tdap vaccine will simply replace Td in existing (Brazil) or planned (Bangladesh, Nigeria) programs, it is unlikely to involve additional delivery costs, although introduction and program-level costs are possible and unknown. The range of prices considered is wide enough to encompass those costs, so they were not estimated separately.

The price of infant DTP vaccine for Bangladesh and Nigeria, $2.23/dose, is the average of all listed 2016 prices per dose for DTP/hepatitis B/*Haemophilus influenzae* type b (Hib) from UNICEF (United Nations Children's Fund) suppliers; it includes wastage (5%), freight, and administrative fees [17–19]. Delivery cost/dose was estimated from the comprehensive multiyear plans of Bangladesh ($0.74) and Nigeria ($5.83) [20]. The price of infant DTP vaccine for Brazil, $2.30, is from the Pan American Health Organization's 2015 price list [16], and includes freight and insurance (3%) and wastage (5%); the delivery cost, $5.97/dose, is from costing studies of routine immunization programs in Honduras and Colombia [21, 22].

The cost of treating fatal pertussis in Bangladesh and Nigeria was estimated by multiplying the World Health Organization (WHO)-CHOICE cost of a hospital bed-day at a secondary level hospital [23] times length of stay in LMICs from an unpublished systematic literature review conducted by the authors (range, 6.0–9.2 days), then adding a percentage for costs of procedures, diagnostic tests, and drugs based on [29]. For Brazil the cost, $1124 per death in 2014, was the reimbursement paid to hospitals by the Public National Healthcare System for infants who died of pertussis [12].

Cost-Effectiveness Benchmarks

The World Health Organization has suggested that an intervention be considered very cost-effective if cost per disability-adjusted life-year (DALY) averted is less than GDP per capita (GDPpc) and cost-effective if cost/DALY is 1–3 times GDPpc [30], although these recommendations are undergoing revision. A recent analysis for the United Kingdom concluded that WHO's guidelines may be too high, encouraging adoption of interventions that displace existing services that provide more health; the authors suggest that 0.51 GDPpc is a more appropriate benchmark for low-income countries and 0.71 GDPpc for middle-income countries [31]. The cost per DALY of vaccines currently delivered to infants and children in LMICs offers another guide. We show results for 3 benchmarks of cost-effectiveness: $100/DALY, the strictest guide from a recent systematic review of vaccine cost-effectiveness [32], 0.5 GDPpc, and GDPpc.

Calculation of Pertussis Mortality Thresholds

For each cost-effectiveness benchmark and vaccine price, the mortality threshold was estimated by running a 1-way sensitivity analysis to identify the infant pertussis mortality rate that produced that benchmark (eg, $100/DALY) at that price in that country.

To estimate an uncertainty interval for each mortality threshold, we ran a probabilistic sensitivity analysis (PSA), holding vaccine price at the price used to derive the threshold, but

Downloaded from http://cid.oxfordjournals.org/ by guest on November 12, 2016

letting other parameters vary according to the distributions in Table 1. A uniform distribution was used for the mortality threshold itself, with a lower bound of 50% and an upper bound of 150% of the threshold rate. The PSA results were then ranked by their cost-effectiveness ratios, and those with cost-effectiveness ratios within 5% of the benchmark (eg, $95–$105 for a benchmark of $100/DALY) were selected. The minimum and maximum infant pertussis mortality rates associated with those cost-effectiveness ratios provide the bounds of the uncertainty interval shown in the chart.

RESULTS

Figures 2–4 show the mortality thresholds estimated for Bangladesh, Nigeria, and Brazil. The horizontal axis shows the cost-effectiveness benchmarks, $100/DALY, 0.5 GDPpc/DALY, and GDPpc/DALY. The vertical axis shows pertussis deaths per 1000 infants. Each bar shows the mortality threshold for a specific cost-effectiveness benchmark and maternal aP vaccine price. For example, the first bar on Figure 2 shows that to achieve a cost-effectiveness benchmark of $100/DALY at a vaccine price of $0.50/dose, the infant pertussis mortality rate in Bangladesh would need to be 0.272 pertussis deaths per 1000 infants.

Infant Pertussis Mortality Thresholds

The key drivers of cost per DALY are the infant pertussis mortality rate and maternal aP vaccine price. Vaccine price, unknown for the vaccine under development, is set at 5 alternative levels within the range considered most likely for each country—$0.50–$5.00/dose for Bangladesh and Nigeria (countries with access to Global Alliance for Vaccines and Immunization funding) and $4–$12/dose for Brazil.

Because mortality and price are key drivers, the mortality thresholds for $100/DALY are similar across countries for the same price, and would be for any fixed-dollar benchmark. For example, for a price of $1/dose, the mortality threshold is 0.546 pertussis deaths per 1000 infants for Bangladesh and 0.555 deaths per 1000 for Nigeria. For benchmarks based on GDPpc, mortality thresholds differ substantially across countries because GDP per capita differs (Table 1).

The range of GDPpc represented by these countries, together with the fact that mortality thresholds are similar for fixed-dollar benchmarks, means that the information in the charts generalizes beyond the particular country. For example, 0.5 GDPpc in Bangladesh is $544, close to the $500/DALY sometimes used as a benchmark [32]. Thus, for the price range $0.50–$5.00/dose, the mortality thresholds for 0.5 GDPpc in Bangladesh show the approximate mortality rates necessary to make maternal aP immunization cost-effective at $500/DALY in any LMIC.

The uncertainty intervals on the charts show that, when other model parameters are allowed to vary according to the distributions in Table 1, the mortality thresholds necessary to hold cost per DALY within 5% of a given benchmark are in a relatively narrow range around the point estimates.

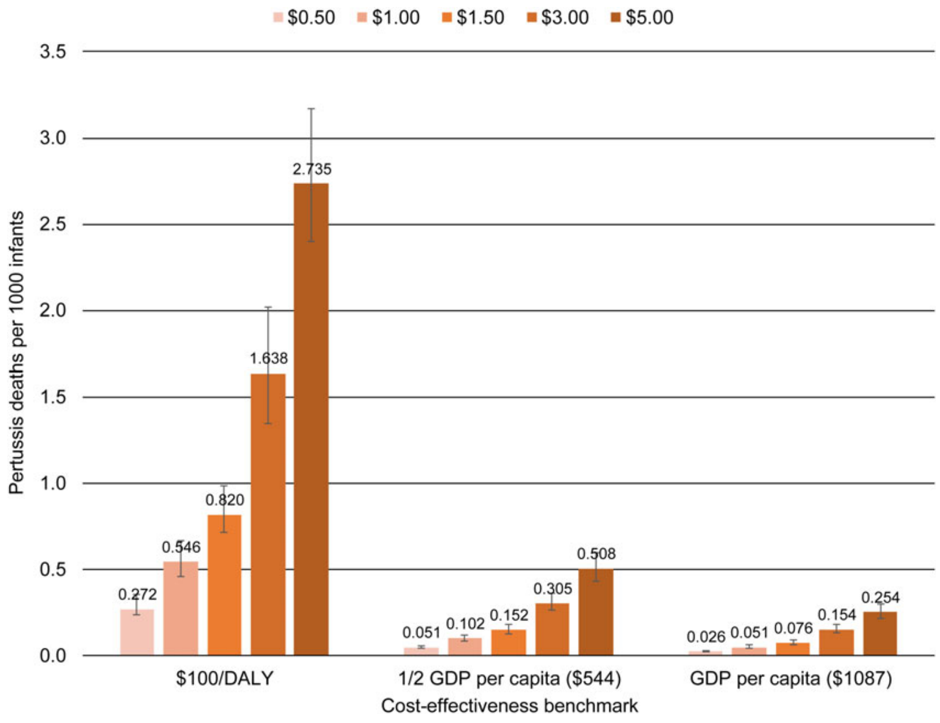

Figure 2. Bangladesh: Pertussis mortality required to make maternal acellular pertussis immunization cost-effective, by vaccine price and cost-effectiveness benchmark. Abbreviations: DALY, disability-adjusted life-year; GDP, gross domestic product.

Downloaded from http://cid.oxfordjournals.org/ by guest on November 12, 2016

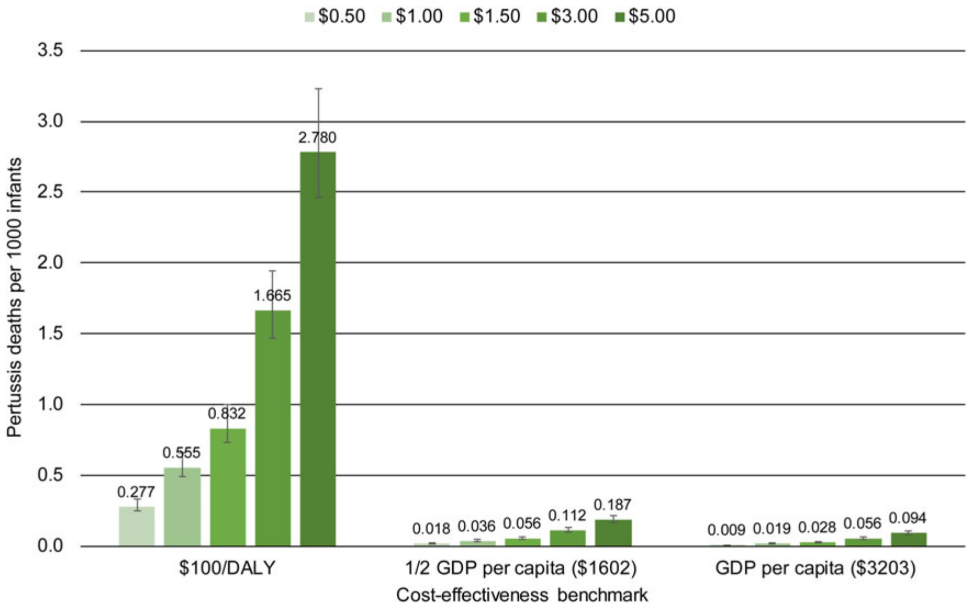

Figure 3. Nigeria: Pertussis mortality required to make maternal acellular pertussis immunization cost-effective, by vaccine price and cost-effectiveness benchmark. Abbreviations: DALY, disability-adjusted life-year; GDP, gross domestic product.

Comparison of Thresholds With Observed Rates

One way to put the mortality thresholds in perspective is to compare them to neonatal and infant mortality in the countries modeled (Table 1). For example, if the aP vaccine costs $5.00/dose, the pertussis mortality rate necessary to make maternal aP immunization cost-effective at $100/DALY in Bangladesh is 2.735 pertussis deaths per 1000 (Figure 2), a rate that would account for 8.5% of infant mortality in that country. These comparisons suggest that the highest pertussis mortality thresholds are unlikely to occur in reality.

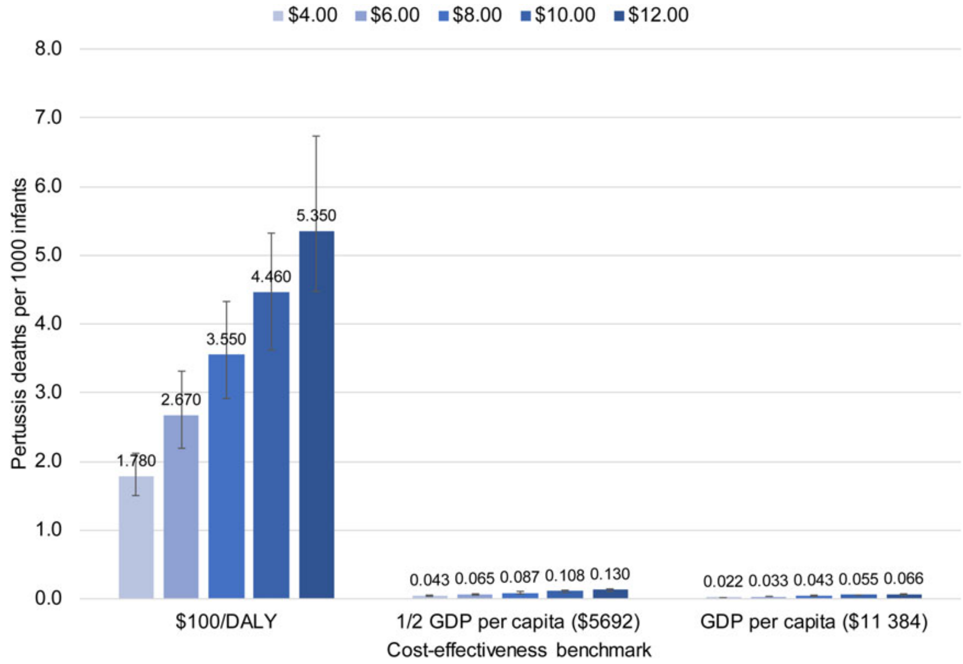

Figure 4. Brazil: Pertussis mortality required to make maternal acellular pertussis immunization cost-effective, by vaccine price and cost-effectiveness benchmark. Abbreviations: DALY, disability-adjusted life-year; GDP, gross domestic product.

Downloaded from http://cid.oxfordjournals.org/ by guest on November 12, 2016

A second perspective is provided by the few available data on pertussis mortality in LMICs. In Brazil most infants with severe pertussis are hospitalized, and data from the Public National Healthcare System [12], which covers 75% of the population, show 0.0254 pertussis deaths per 1000 infants in 2014. Ongoing community-based surveillance studies of pertussis in Pakistan and Zambia, and community-based studies of lower respiratory tract infection that conducted secondary pertussis testing in Asia and Africa, have reported no hospitalizations or deaths due to pertussis [6], suggesting that a similarly low mortality rate may hold in other LMICs. The mortality rates that make maternal aP immunization cost-effective at the selected cost-effectiveness benchmarks are >0.0254 pertussis deaths per 1000 except at the lowest vaccine prices (Figures 2–4). If pertussis mortality equals the Brazilian rate, aP vaccine would have to cost $0.50/dose in Bangladesh, $1.35 in Nigeria, and $4.70 in Brazil for maternal immunization to be cost-effective at GDPpc. To be cost-effective at the lower benchmark of 0.5 GDPpc the price would need to be cut by half: $0.25 in Bangladesh, $0.70 in Nigeria, and $2.35 in Brazil.

A third perspective comes from estimates that pertussis caused 56 700 deaths among children under 5 in 2015, 2700 of the deaths in neonates [33]. If postneonatal deaths are evenly distributed over the remaining 59 months, 12 678 deaths occurred among 126 517 000 infants [34] in 2015, for an infant pertussis mortality rate of 0.1009 per 1000 infants, almost 4 times the Brazilian rate. This rate meets or exceeds a larger number of the mortality thresholds in Figures 2–4 and would allow maternal aP immunization to be cost-effective at higher vaccine prices. At this infant pertussis mortality rate, aP vaccine could cost as much as $2.00/dose in Bangladesh, $5.40 in Nigeria, and $18.80 in Brazil for maternal immunization to be cost-effective at GDPpc. To be cost-effective at the lower benchmark of 0.5 GDPpc, the price would need to be $1.00 in Bangladesh, $2.70 in Nigeria, and $9.40 in Brazil.

Sensitivity Analyses

We conducted 1-way sensitivity analyses on 2 important parameters: maternal immunization coverage and effectiveness.

Whereas differences in maternal aP coverage cause substantial differences in total costs and DALYs averted, they make almost no difference to cost-effectiveness as costs and DALYs increase/decrease in parallel, leaving cost per DALY unchanged. Thus the results reported here would not change if alternative measures of coverage were used, such as the proportion of pregnant women with at least 2 doses of tetanus toxoid.

If aP vaccine efficacy were <85%, our base case assumption, vaccine price would need to be lower to achieve a given cost-effectiveness benchmark. Setting infant pertussis mortality at the Brazilian rate, and vaccine effectiveness at 70%, aP vaccine would need to cost $0.40/dose in Bangladesh, $1.15 in Nigeria, and $3.85 in Brazil to be cost-effective at GDPpc. At the higher infant pertussis mortality rate based on [33], the vaccine could cost $1.65/dose in Bangladesh, $4.45 in Nigeria, and $15.40 in Brazil to be cost-effective at GDPpc.

Finally, we note that UNICEF has negotiated substantially lower infant vaccine prices for 2017–2018 [35]. Although the lower prices will reduce the cost of routine infant vaccination, they will make virtually no difference to the cost-effectiveness of maternal aP immunization, and thus to the mortality thresholds, as routine infant vaccination is included in both strategies.

DISCUSSION

This analysis focuses on the potential contribution of maternal aP immunization to Sustainable Development Goal 3's target of ending preventable deaths of infants and children <5 years of age in LMICs. Using a decision model, we estimated the threshold infant pertussis mortality rates necessary to make maternal aP immunization cost-effective in LMICs for selected cost-effectiveness benchmarks and a range of vaccine prices. In many cases the necessary mortality rates exceed the rates known or considered likely. Our findings suggest that maternal aP vaccination approaches cost-effectiveness only when the vaccine price is low, less than $1–$2/dose, or the cost-effectiveness benchmark is at the high end of those considered in this report (GDP per capita).

The pertussis mortality rate in infants is critical to this conclusion, but good information on infant pertussis mortality rates is sparse and estimates range widely. Globally, a recent study estimated 56 700 pertussis deaths among children <5 years of age in 2015, 2700 in neonates, an approximate risk of 0.1 pertussis deaths per 1000 infants [33]. Brazil, an upper-middle-income country, reports a rate of 0.0254 pertussis deaths per 1000 infants (see Methods). As the Brazilian rate indicates, global averages may not apply to individual countries where pertussis mortality risk may be higher or lower depending on demography, childhood vaccine coverage, pertussis transmission patterns, child nutrition, concomitant morbidity, and access to care [36].

Other cost-effectiveness studies have reached similar conclusions. A Brazilian study found that, at a vaccine cost of $12.39/dose and a discount rate of 3%, maternal aP immunization would cost about $40 000 per life-year saved (in 2011 dollars), a cost that exceeds 3 times Brazil's GDP per capita [3]. A US study, which used a vaccine cost of $37.60/dose, estimated that maternal aP immunization would cost almost $500 000 per life-year (2011$) [37]. An English evaluation concluded that maternal immunization's cost-effectiveness depends critically on incidence; it would cost £16 685 ($24 162) per quality-adjusted life-year if pertussis incidence continues at the English high of 2012, but would be substantially more expensive if instead it remains at the lowest levels of recent years [38].

There is no consensus on exactly how much to spend to avert a DALY, especially in LMICs. To provide decision makers with

Downloaded from http://cid.oxfordjournals.org/ by guest on November 12, 2016

some guidance, the WHO initially proposed 2 benchmarks: 1 time and 3 times GDP per capita per DALY averted [30]. Recent work, however, suggests that these benchmarks may be too high, and using them for decision making runs the risk of accepting new interventions that may crowd out other interventions that provide more health; it has been suggested that half of GDP per capita per DALY may be a better benchmark [31], although further work in this field is needed. In the poorest countries, half GDP per capita can be $500 or less per DALY. In the 3 countries analyzed in this study, half GDP per capita is $544 in 2014 US dollars (Bangladesh), $1602 (Nigeria), and $5692 (Brazil). As another way to put these benchmarks in perspective, a systematic review of cost-effectiveness studies of childhood vaccines used in LMICs found that hepatitis B, Hib, measles, and tuberculosis vaccines cost less than $100/DALY in many LMICs, while pneumococcal and rotavirus vaccines often cost less than $500 or $1000/DALY [32] (Table 1). Thus, most vaccines currently delivered to infants and children in LMICs, including newer vaccines that are more costly than the traditional vaccines used in routine immunization schedules, offer better value than would maternal aP immunization.

Not all decision makers will want to focus solely on mortality. In middle-income countries, where child mortality is low, averting morbidity, outpatient care, and hospitalizations may also be important public health goals. Including these outcomes in a cost-effectiveness analysis would increase DALYs averted, although only slightly as pertussis morbidity is short-lived, and reduce treatment costs, offsetting part of the cost of maternal aP immunization. Thus, including these outcomes would improve the program's cost-effectiveness.

Our analyses show, however, that where the primary objective is to prevent deaths, maternal aP immunization is likely to be cost-effective in low-income countries only if the vaccine price can be reduced below $1/dose, except in countries where the infant mortality rate from pertussis is high.

Supplementary Data

Supplementary materials are available at http://cid.oxfordjournals.org. Consisting of data provided by the author to benefit the reader, the posted materials are not copyedited and are the sole responsibility of the author, so questions or comments should be addressed to the author.

Notes

Acknowledgments. We thank Colin Sanderson for providing his analyses of Demographic and Health Surveys data pertaining to vaccine coverage; Fatimah Abdul-Hakeem for her help developing the delivery costs for infant vaccination in Bangladesh and Nigeria; and Erin Andrews and Sun-Young Kim for their comments on a draft of this manuscript. We also thank the Brazilian Ministry of Health for providing surveillance and vaccine coverage data and contributing to the interpretation of pertussis data in Brazil.

Author contributions. L. B. R., A. S., and C. M. T. were primarily responsible for the conception and design of the work. All authors participated in the acquisition, analysis, and interpretation of data for the work; in drafting the work or revising it critically for important intellectual content; gave final approval of the version to be published; and agree to be accountable for all aspects of the work in ensuring that questions related to the accuracy or integrity of any part of the work are appropriately investigated and resolved.

Financial support. This work was supported by the Bill & Melinda Gates Foundation (grant number OPP1124529).

Supplement sponsorship. This article appears as part of the supplement "Infant Pertussis Disease Burden in the Context of Maternal Immunization Strategies," sponsored by the Bill & Melinda Gates Foundation.

Potential conflicts of interest. All authors: No reported conflicts. All authors have submitted the ICMJE Form for Disclosure of Potential Conflicts of Interest. Conflicts that the editors consider relevant to the content of the manuscript have been disclosed.

References

1. United Nations. Sustainable Development Goals: 17 goals to transform our world. Available at: http://www.un.org/sustainabledevelopment/health/. Accessed 20 April 2016.
2. Vizzotti C, Neyro S, Katz N, et al. Maternal immunization in Argentina: a storyline from the prospective of a middle income country. Vaccine **2015**; 33:6413–9.
3. Sartori AM, de Soarez PC, Fernandes EG, Gryninger LC, Viscondi JY, Novaes HM. Cost-effectiveness analysis of universal maternal immunization with tetanus-diphtheria-acellular pertussis (Tdap) vaccine in Brazil. Vaccine **2016**; 34:1531–9.
4. Amirthalingam G, Andrews N, Campbell H, et al. Effectiveness of maternal pertussis vaccination in England: an observational study. Lancet **2014**; 384:1521–8.
5. US Centers for Disease Control and Prevention. Get the whooping cough vaccine while you are pregnant. Available at: http://www.cdc.gov/pertussis/pregnant/mom/get-vaccinated.html. Accessed 3 June 2016.
6. Bill & Melinda Gates Foundation. Pertussis mini-symposium. Atlanta, GA, 23 February 2016.
7. United Nations. Live births, deaths, and infant deaths, latest available year (2000–2014). Available at: http://unstats.un.org/unsd/demographic/products/vitstats/serATab3.pdf. Accessed 1 June 2016.
8. United Nations Inter-agency Group for Child Mortality Estimation. Child mortality estimates. Available at: http://www.childmortality.org/. Accessed 26 October 2015.
9. United Nations, Department of Economic and Social Affairs, Population Division (2015). World population prospects: the 2015 revision, DVD edition. File MORT/16-1: life expectancy at exact age, e(x), for both sexes combined, by major area, region and country, 1950-2100. Available at: https://esa.un.org/unpd/wpp/Download/Standard/Mortality/. Accessed 23 February 2015.
10. United Nations, Department of Economic and Social Affairs, Population Division (2015). World population prospects: the 2015 revision, DVD edition. File MORT/15-1: Life table survivors at exact age, l(x), for both sexes combined, by major area, region and country, 1950–2100. Available at: https://esa.un.org/unpd/wpp/Download/Standard/Mortality/. Accessed 23 February 2015.
11. World Bank national accounts data, and OECD National Accounts data files. World development indicators. GDP per capita (current US$). Available at: http://data.worldbank.org/indicator/NY.GDP.PCAP.CD. Accessed 28 March 2016.
12. Brasilian DATASUS. Sistema de Informação de Hospitalizações (SIH-SUS). SUS hospital morbidity. CID-10 Pertussis. Hospitalizations by year service, by place of residence, Brazil, **2014**. Available at: http://tabnet.datasus.gov.br/cgi/tabcgi.exe?sih/cnv/nruf.def. Accessed 6 February 2016.
13. Juretzko P, von Kries R, Hermann M, Wirsing von Konig CH, Weil J, Giani G. Effectiveness of acellular pertussis vaccine assessed by hospital-based active surveillance in Germany. Clin Infect Dis **2002**; 35:162–7.
14. National Institute of Population Research and Training (NIPORT), Mitra and Associates, and ICF International.2016. Bangladesh Demographic and Health Survey 2014. Dhaka, Bangladesh, and Rockville, Maryland: NIPORT, Mitra and Associates, and ICF International. Available at: https://dhsprogram.com/pubs/pdf/FR311/FR311.pdf. Accessed 17 May 2016.
15. National Population Commission (NPC) [Nigeria] and ICF International. 2014. Nigeria Demographic and Health Survey 2013. Abuja, Nigeria, and Rockville, Maryland, USA: NPC and ICF International. Available at: https://dhsprogram.com/pubs/pdf/FR293/FR293.pdf. Accessed 17 May 2016.
16. Pan American Health Organization. Maternal and infant vaccine prices for Brazil from the Pan American Health Organization Revolving Fund 2015 price list. Available at: http://www.paho.org. Accessed 16 December 2015.
17. United Nations Children's Fund. Supplies and logistics. Vaccine price data. Available at: http://www.unicef.org/supply/index_57476.html. Accessed 19 November 2015.

Downloaded from http://cid.oxfordjournals.org/ by guest on November 12, 2016

18. United Nations Children's Fund. Supplies and logistics. Auto-disable (AD) and Re-use prevention (RUP) syringes and safety boxes—current price data. Available at: http://www.unicef.org/supply/index_62309.html. Accessed 11 March 2016.
19. United Nations Children's Fund. Supplies and logistics. Procurement services. Available at: http://www.unicef.org/supply/index_procurement_services.html. Accessed 11 March 2016.
20. World Health Organization. The cMYP immunization financing database. Available at: http://www.who.int/immunization/programmes_systems/financing/countries/en/. Accessed 28 September 2015.
21. Janusz CB, Castañeda-Orjuela C, Molina Aguilera IB, et al. Examining the cost of delivering routine immunization in Honduras. Vaccine **2015**; 33(suppl 1):A53–9.
22. Castañeda-Orjuela C, Romero M, Arce P, et al. Using standardized tools to improve immunization costing data for program planning: the cost of the Colombian Expanded Program on Immunization. Vaccine **2013**; 31(suppl 3):C72–9.
23. World Health Organization. Choosing interventions that are cost effective (WHO-CHOICE). Country-specific unit costs. Available at: http://www.who.int/choice/country/country_specific/en/. Accessed 30 September 2015.
24. Clark A, Sanderson C. Timing of children's vaccinations in 45 low-income and middle-income countries: an analysis of survey data. Lancet **2009**; 373:1543–9.
25. World Health Organization. Immunization schedules by antigens—World Health Organization. Available at: http://apps.who.int/immunization_monitoring/globalsummary/schedules. Accessed 21 January 2016.
26. Van Rie A, Wendelboe AM, Englund JA. Role of maternal pertussis antibodies in infants. Pediatr Infect Dis J **2005**; 24(5 suppl):S62–5.
27. World Bank. World development indicators. Inflation, GDP deflator (annual %). Available at: http://data.worldbank.org/indicator/NY.GDP.DEFL.KD.ZG. Accessed 31 March 2016.
28. International Monetary Fund, International Financial Statistics. World Development Indicators. Official exchange rate (LCU per US$, period average). Available at: http://data.worldbank.org/indicator/PA.NUS.FCRF. Accessed 31 March 2016.
29. Sinha A, Kim S, Ginsberg G, et al. Economic burden of acute lower respiratory tract infection in South African children. Paediatr Int Child Health **2012**; 32:65–73.
30. Marseille E, Larson B, Kazi DS, Kahn JG, Rosen S. Thresholds for the cost-effectiveness of interventions: alternative approaches. Bull World Health Organ **2015**; 93:118–24.
31. Woods B, Revill P, Sculpher M, Claxton K. Country-level cost-effectiveness thresholds: initial estimates and the need for further research. In: CHE Research Paper 109. March 2015. Centre for Health Economics, University of York. Available at: www.york.ac.uk/che/publications/. Accessed 7 April 2016.
32. Ozawa S, Mirelman A, Stack ML, Walker DG, Levine OS. Cost-effectiveness and economic benefits of vaccines in low- and middle-income countries: a systematic review. Vaccine **2012**; 31:96–108.
33. Liu L, Oza S, Hogan D, et al. Global, regional and national causes of child mortality in 2000–2015—implications for the Sustainable Development Goals. Lancet **2016**. In press.
34. US Census Bureau. World population by age and sex. Available at: http://www.census.gov/population/international/data/idb/worldpop.php. Accessed 31 August 2016.
35. UNICEF, 2016_05_24_DTP-HepB-Hib-awarded_prices. Available at: http://www.unicef.org/supply/files/2016_05_24_DTP-HepB-Hib-awarded_prices_24_May.pdf. Accessed 30 June 2016.
36. Edwards KM, Decker MD. Pertussis vaccines. In: Plotkin SM, Orenstein WA, Offit PA. Vaccines. 6th ed. New York: Elsevier Saunders, **2013**:447–92.
37. Terranella A, Beeler Asay GR, Messonnier M, Clark TA, Liang JL. Pregnancy dose Tdap and postpartum cocooning to prevent infant pertussis: a decision analysis. Pediatrics **2013**; 131:e1748–56.
38. van Hoek AJ, Campbell H, Amirthalingam G, Andrews N, Miller E. Cost-effectiveness and programmatic benefits of maternal vaccination against pertussis in England. J Infect **2016**; 73:28–37.

Downloaded from http://cid.oxfordjournals.org/ by guest on November 12, 2016
